# Antimicrobial activity of ceftobiprole and comparator agents when tested against gram-positive and -negative organisms collected across China (2016–2018)

**DOI:** 10.1186/s12866-022-02699-4

**Published:** 2022-11-26

**Authors:** Yin Dandan, Wu Shi, Yang Yang, Zheng Yonggui, Demei Zhu, Guo Yan, Fupin Hu

**Affiliations:** 1 Institute of Antibiotics, Huashan Hospital, Fudan University, 12 M. Wulumuqi Rd, Shanghai, 200040 People’s Republic of China; 2 Key Laboratory of Clinical Pharmacology of Antibiotics, Ministry of Health, Shanghai, China

**Keywords:** *S. aureus*, *E. faecalis*, *H. influenzae*, *Streptococcus spp*, *M. catarrhalis*, *E. coli*, *K. pneumoniae*, Ceftobiprole, Minimal inhibitory concentration

## Abstract

**Background:**

Ceftobiprole is a fifth-generation cephalosporin which has been reported to have broad antibacterial spectrum when tested against bacteria collected from other countries except China. This study evaluated the in vitro activity of ceftobiprole in comparison with other comparators against clinically significant isolates collected across from China.

**Results:**

Susceptibility testing of ceftobiprole and comparators against 1163 clinically isolated Gram-positive and Gram-negative bacteria was performed with broth micro dilution method following the CLSI guidelines. All 110 *S. aureus* were susceptible to ceftobiprole with MIC_50/90_ of 1/2 mg/L for MRSA and 0.5/1 mg/L for MSSA. For Coagulase-negative *staphylococci* (CNS), MIC_50/90_ of ceftobiprole for MRCNS and MSCNS was 1/2 mg/L and 0.25/0.5 mg/L. Ceftobiprole demonstrated good potency against *E. faecalis* (MIC_50/90_ of 0.5/1 mg/L) but limited activity against *E. faecium* (MIC_50/90_ of > 32/ > 32 mg/L). Ceftobiprole demonstrated potent activity against all 39 β-hemolytic *Streptococcus spp.* with MIC_50/90_ ≤ 0.015/ ≤ 0.015–2 mg/L and 110 of PSSP with 98.2% susceptibility. Ceftobiprole inhibited all isolates of *H. influenzae* and *M. catarrhalis* at ≤ 1 mg/L. 91.8% and 98.2% of the ESBL-negative *E. coli* and *K. pneumoniae* were susceptible to ceftobiprole, but most of the ESBL-positive or carbapenem-resistant strains were also resistant to ceftobiprole. Ceftobiprole inhibited 84.2% of carbapenem-susceptible *P. aeruginosa* and 94.1% of carbapenem-susceptible *A. baumannii* at ≤ 8 mg/L, but only 52.6% of carbapenem-resistant *P. aeruginosa* and 5.3% of carbapenem-resistant *A. baumannii*.

**Conclusion:**

Ceftobiprole demonstrated good in vitro activity against a broad range of clinically relevant contemporary Gram-positive and Gram-negative bacterial isolates.

## Background

Antimicrobial resistance has been a public health threat in recent years, with an increase of multi-drug resistant bacteria, such as extended-spectrum β-lactamase positive *Enterobacterales*, methicillin-resistant *Staphylococcus aureus* (MRSA), Vancomycin-resistant *E. faecium* and penicillin-non-susceptible *S. pneumoniae* (PRSP), which are listed as the important pathogens for new antibiotics by WHO [1]. Ceftobiprole is a fifth-generation parenteral cephalosporin demonstrating potent in vitro activity against Gram-positive pathogens, including MRSA and PRSP, as well as some non-carbapenemase or ESBL-producing Gram-negative pathogens commonly associated with pneumonia [2, 3]. It has obtained regulatory approval in Europe and several non-European countries for the treatment of hospital-acquired pneumonia excluding ventilator-associated pneumonia and community-acquired pneumonia in adults [4, 5]. It has been reported that ceftobiprole is generally β-lactamase stable and has a strong affinity for essential penicillin-binding proteins, including those responsible for β-lactam resistance in staphylococci and pneumococci [6]. Several studies have been reported on the spectrum and potency of ceftobiprole against Gram-positive and Gram-negative pathogens collected from Europe and surrounding countries in a variety of infection types [2–4, 7, 8]. In this present study, we expand upon those observations by reporting the activity of ceftobiprole and comparators against bacterial isolates obtained and tested during the 2016–2018 CHINET Antimicrobial Surveillance Network in China.

## Results

### Ceftobiprole and comparator antibiotics activity against gram-positive bacteria

Ceftobiprole was active against 110 *S. aureus* (MIC range, 0.25–2 mg/L, 100% susceptibility) and 80 Coagulase-negative *Staphylococci* (CNS, MIC range, ≤ 0.015—4 mg/L). All *S. aureus* and CNS were susceptible to vancomycin and linezolid. For MRSA, susceptibility to ciprofloxacin, clindamycin, and erythromycin was 54.5%, 23.6%, and 12.7%, which was less than that of methicillin-susceptible *S. aureus* (MSSA), 83.6%, 72.7%, and 43.6%, respectively. Ceftobiprole was twice as active against MSSA strains with MIC_50/90_ of 0.5/1 mg/L than on MRSA strains with MIC_50/90_ of 1/2 mg/L. For methicillin-resistant Coagulase-negative *Staphylococci* (MRCNS), ciprofloxacin, clindamycin, and erythromycin susceptibility were 17.5%, 57.5%, and 12.5%, which were all less than that of methicillin-susceptible Coagulase-negative *Staphylococci* (MSCNS), 67.5%, 85%, and 32.5%, respectively. Ceftobiprole was two-fold more active on MSCNS strains with MIC_50/90_ of 0.25/0.5 mg/L than on MRCNS strains with MIC_50/90_ of 1/2 mg/L (Table 1).

**Table 1:** Activity of ceftobiprole and comparator antimicrobial agents when tested against *Staphylococcus* isolated from China (mg/L)

**Antimicrobial agents**	**MIC Range**	**MIC**_**50**_	**MIC**_**90**_	**R%**	**S%**
**Methicillin-resistant *Staphylococcus aureus* (MRSA) (55)**					
Ceftobiprole	0.25 – 2	1	2	0	100
Linezolid	0.25 – 2	0.5	1	0	100
Vancomycin	0.25 – 1	0.5	1	0	100
Penicillin	4 – > 32	> 32	> 32	100	0
Oxacillin	4 – > 4	> 4	> 4	100	0
Ciprofloxacin	0.25 – > 32	1	> 32	45.5	54.5
Clindamycin	≤ 0.06 – > 128	> 128	> 128	76.4	23.6
Erythromycin	0.125 – > 128	> 128	> 128	85.5	12.7
**Methicillin-susceptible *Staphylococcus aureus* (MSSA) (55)**					
Ceftobiprole	0.25 – 2	0.5	1	0	100
Linezolid	0.25 – 1	0.5	1	0	100
Vancomycin	0.25 – 1	0.5	1	0	100
Penicillin	0.03 – > 32	8	32	85.5	14.5
Oxacillin	≤ 0.25 – 1	≤ 0.25	0.5	0	100
Ciprofloxacin	0.25 – 32	0.5	16	12.7	83.6
Clindamycin	≤ 0.06 – > 128	0.125	> 128	23.6	72.7
Erythromycin	0.125 – > 128	> 128	> 128	56.4	43.6
**Methicillin-resistant Coagulase negative *Staphylococci* (MRCNS) (40)**					
Ceftobiprole	≤ 0.015 – 4	1	2	–	–
Linezolid	0.5 – 4	1	1	0	100
Vancomycin	0.5 – 2	1	2	0	100
Penicillin	0.5 – > 32	16	> 32	100	0
Oxacillin	0.5 – > 4	4	> 4	100	0
Ciprofloxacin	0.125 – > 32	16	> 32	77.5	17.5
Clindamycin	≤ 0.06 – > 128	≤ 0.06	> 128	40	57.5
Erythromycin	≤ 0.06 – > 128	> 128	> 128	87.5	12.5
**Methicillin-susceptible Coagulase negative *Staphylococci* (MSCNS) (40)**					
Ceftobiprole	≤ 0.015 – 1	0.25	0.5	–	–
Linezolid	0.5 – 2	0.5	1	0	100
Vancomycin	0.5 – 2	1	2	0	100
Penicillin	≤ 0.015 – > 32	0.25	8	65	35
Oxacillin	≤ 0.25	≤ 0.25	≤ 0.25	0	100
Ciprofloxacin	0.125 – 16	0.25	4	15	67.5
Clindamycin	≤ 0.06 – > 128	≤ 0.06	> 128	15	85
Erythromycin	≤ 0.06 – > 128	64	> 128	67.5	32.5

Ceftobiprole was also active against 24 *E. faecalis* with MIC_50/90_ of 0.5/1 mg/L but showed no clinically relevant activity against 24 *E. faecium* with both MIC_50_ and MIC_90_ > 32 mg/L. All *E. faecium* were susceptible to vancomycin and linezolid, but 8.3% of *E. faecalis* was intermediate to linezolid. For *E. faecalis*, the resistance rate to ampicillin, ciprofloxacin, and erythromycin was much less than that for *E. faecium* (8.3%, 29.2%, and 62.5% VS 82.6%, 87%, and 91.3%) (Table 2).

**Table 2:** Activity of ceftobiprole and comparator antimicrobial agents when tested against *Enterococcus* isolated from China (mg/L)

**Antimicrobial agents**	**MIC Range**	**MIC**_**50**_	**MIC**_**90**_	**R%**	**S%**
***Enterococcus faecalis* (24)**					
Ceftobiprole	0.06 – > 32	0.5	1	–	–
Linezolid	0.5 – 4	0.5	2	0	91.7
Vancomycin	0.5 – 2	0.5	1	0	100
Ampicillin	1 – > 128	1	4	8.3	91.7
Ciprofloxacin	0.25 – > 32	1	> 32	29.2	70.8
Erythromycin	1 – > 128	> 128	> 128	62.5	0
***Enterococcus faecium* (23)**					
Ceftobiprole	0.5 – > 32	> 32	> 32	–	–
Linezolid	0.25 – 1	0.5	0.5	0	100
Vancomycin	0.25 – 4	0.5	0.5	0	100
Ampicillin	1 – > 128	> 128	> 128	82.6	17.4
Ciprofloxacin	1 – > 32	> 32	> 32	87	8.7
Erythromycin	0.125 – > 128	> 128	> 128	91.3	4.3

Ceftobiprole demonstrated good activity against PSSP (susceptibility of 98.2%), which was similar to linezolid and vancomycin, whereas only half of the PISP and PRSP were susceptible to it. Erythromycin showed poor activity against all *S. pneumoniae*. Ceftobiprole demonstrated potent activity against all 39 *Streptococcus* with MIC_50/90_ ≤ 0.015/ ≤ 0.015–2 mg/L, which is far better than that of linezolid and vancomycin (both MIC_50/90_ are 0.25/0.25–0.5 mg/L). All 13 *Streptococcus pyogenes* were resistant to erythromycin, while 35.7% of *Streptococcus agalactiae* and 33.3% of *Streptococcus mitis* remained susceptible to it (Table 3).

**Table 3:** Activity of ceftobiprole and comparator antimicrobial agents when tested against *Streptococcus* isolated from China (mg/L)

**Antimicrobial agents**	**MIC Range**	**MIC**_**50**_	**MIC**_**90**_	**R%**	**S%**
***Streptococcus pyogenes* (13)**					
Ceftobiprole	≤ 0.015 – ≤ 0.015	≤ 0.015	≤ 0.015	–	–
Linezolid	0.25 – 0 .25	0.25	0.25	0	100
Vancomycin	0.125 – 0 .25	0.25	0.25	0	100
Penicillin	≤ 0.015 – 0.06	≤ 0.015	0.03	0	100
Ciprofloxacin	0.125 – 1	0.25	0.25		
Erythromycin	64 – > 128	> 128	> 128	100	0
***Streptococcus agalactiae* (14)**					
Ceftobiprole	≤ 0.015 – ≤ 0.015	≤ 0.015	≤ 0.015	–	–
Linezolid	0.25 – 0.5	0.25	0.5	0	100
Vancomycin	0.25 – 0 .25	0.25	0.25	0	100
Penicillin	≤ 0.015 – 0.125	0.03	0.125	0	100
Ciprofloxacin	0.25 – 16	0.5	16	–	–
Erythromycin	≤ 0.06 – > 128	> 128	> 128	64.3	35.7
***Streptococcus mitis* (12)**					
Ceftobiprole	≤ 0.015 – 2	≤ 0.015	2	–	–
Linezolid	≤ 0.06 – 0.5	0.25	0.25	0	100
Vancomycin	0.25 – 0.5	0.25	0.5	0	100
Penicillin	≤ 0.015 – 2	0.06	2	0	58.3
Ciprofloxacin	0.5 – 32	2	4	–	–
Erythromycin	≤ 0.06 – > 128	1	> 128	66.7	33.3
***Streptococcus pneumonia* (MIC of Penicillin** ≤ **2 mg/L) (PSSP) (110)**					
Ceftobiprole	≤ 0.015 – 1	0.125	0.5	1.8	98.2
Linezolid	≤ 0.06 – 2	0.5	1	0	100
Vancomycin	≤ 0.06 – 0.25	0.125	0.25	0	100
Penicillin	≤ 0.015 – 2	0.5	2	0	100
Ciprofloxacin	0.03 – 16	0.5	1	–	–
Erythromycin	≤ 0.06 – > 128	> 128	> 128	90.9	5.5
***Streptococcus pneumonia* (MIC of Penicillin = 4 mg/L) (PISP) (25)**					
Ceftobiprole	0.125 – 1	0.5	1	48	52
Linezolid	0.125 – 2	0.5	1	0	100
Vancomycin	≤ 0.06 – 0.25	0.25	0.25	0	100
Penicillin	4 – 4	4	4	0	0
Ciprofloxacin	0.25 – 2	1	2	–	–
Erythromycin	2 – > 128	> 128	> 128	100	0
***Streptococcus pneumonia* (MIC of Penicillin** ≥ **8 mg/L) (PRSP) (13)**					
Ceftobiprole	0.5 – 32	0.5	2	46.2	53.8
Linezolid	0.125 – 1	0.25	1	0	100
Vancomycin	0.125 – 0.25	0.125	0.25	0	100
Penicillin	8 – 32	8	16	100	0
Ciprofloxacin	0.5 – 8	1	4	–	–
Erythromycin	4 – > 128	> 128	> 128	100	0

### Ceftobiprole and comparator antibiotics activity against gram-negative bacteria

Ceftobiprole exhibited potent activity against *Haemophilus influenzae* (MIC_50/90_, ≤ 0.015/0.5 mg/L). Ceftobiprole also showed good activity against *Moraxella catarrhalis* with MIC_50/90_ of 0.25/0.5 mg/L. All *H. influenzae* and *M. catarrhalis* were inhibited at MIC of ≤ 1 mg/L ceftobiprole, and highly susceptible to ampicillin-sulbactam, cefuroxime, ceftazidime, ceftriaxone, and ciprofloxacin with susceptibility rates ranged from 63.9% to 100% (Table 4).

**Table 4:** Activity of ceftobiprole and comparator antimicrobial agents when tested against *Haemophilus influenzae* and *Moraxella catarrhalis* isolated from China (mg/L)

**Antimicrobial agents**	**MIC Range**	**MIC**_**50**_	**MIC**_**90**_	**R%**	**S%**
***Haemophilus influenzae* (53)**					
Ceftobiprole	≤ 0.015 – 1	≤ 0.015	0.5	–	–
Ampicillin	0.03 – 32	1	32	44.2	51.9
Ampicillin-Sulbactam	0.06 – 4	1	2	5.8	94.2
Cefuroxime	0.25 – 16	1	2	1.9	98.1
Ceftazidime	≤ 0.015 – 2	0.06	0.5	0	100
Ceftriaxone	≤ 0.015 – .5	≤ 0.015	0.25	0	100
Ciprofloxacin	≤ 0.015 – 4	≤ 0.015	0.5	1.9	98.1
Azithromycin	≤ 0.015 – > 32	1	> 32	40.4	59.6
***Moraxella catarrhalis* (49)**					
Ceftobiprole	0.06 – 1	0.25	0.5	–	–
Ampicillin	0.5 – 32	2	16	4.1	89.8
Ampicillin-Sulbactam	0.06 – 0 .5	0.125	0.25	0	100
Cefuroxime	0.125 – 8	2	4	0	93.9
Ceftazidime	0.06 – 0.25	0.06	0.25	0	100
Ceftriaxone	0.06 – 2	0.5	2	0	100
Ciprofloxacin	≤ 0.015 – 1	0.06	0.5	0	100
Azithromycin	0.06 – > 32	1	> 32	67.3	32.7

Ceftobiprole had limited activity (0% and 6.9% susceptible) against most ESBL-producers, in contrast to a susceptibility rate of 91.8% and 98.2% found against non-ESBL *E. coli* and *K. pneumoniae.* For non-ESBL strains, the potency of ceftobiprole was similar to ceftazidime, ceftriaxone, cefoperazone-sulbactam, imipenem, amikacin, colistin, and tigecycline, but against ESBL-producers, ceftobiprole performed worse than these other cephalosporins. Ceftobiprole also showed no activity against carbapenem-resistant *K. pneumoniae* (MIC_50/90_, > 128/ > 128 mg/L), some of which were susceptible to amikacin (40%), colistin (91.1%), and tigecycline (100%). Ceftobiprole showed moderate activity against *E. aerogenes, C. freudii, P. mirabilis,* and *M. morganella*, with over 50% of strains inhibited at ≤ 0.06 mg/L. For *E. cloacae* and *S. marcescens*, over 50% of strains were inhibited at 0.25–0.5 mg/L. Ceftobiprole had little activity against *P. vulgaris*, with MIC_50/90_ of 32/ > 128 mg/L (Table 5a-c).

**Table 5:** Activity of ceftobiprole and comparator antimicrobial agents when tested against *Enterobacteriaceae* isolated from China (mg/L)

**Antimicrobial agents**	**MIC Range**	**MIC**_**50**_	**MIC**_**90**_	**R%**	**S%**
**a**					
***Escherichia coli* (ESBL-) (49)**					
Ceftobiprole	≤ 0.06 – 2	≤ 0.06	0.25	8.2	91.8
Ceftazidime	≤ 0.06 – 2	0.25	2	0	100
Ceftriaxone	≤ 0.06 – 8	≤ 0.06	0.25	2	98
Cefoperazone-Sulbactam	≤ 0.06 – 32	0.5	8	0	98
Imipenem	≤ 0.06 – 0.25	0.125	0.125	0	100
Amikacin	0.5 – > 128	1	4	2	98
Colistin	0.25 – 4	0.5	0.5	2	98
Tigecycline	0.125 – 1	0.25	0.5	0	100
***Escherichia coli* (ESBL +) (50)**					
Ceftobiprole	1 – > 128	> 128	> 128	100	0
Ceftazidime	4 – > 128	16	128	64	14
Ceftriaxone	4 – > 128	> 128	> 128	100	0
Cefoperazone-Sulbactam	2 – > 128	16	64	20	58
Imipenem	≤ 0.06 – 0.5	0.125	0.25	0	100
Amikacin	0.5 – > 128	2	128	12	88
Colistin	0.25 – 4	0.5	1	4	96
Tigecycline	0.125 – 1	0.25	0.5	0	100
***Klebsiella pneumonia* (ESBL-) (56)**					
Ceftobiprole	≤ 0.06 – > 128	≤ 0.06	0.25	1.8	98.2
Ceftazidime	≤ 0.06 – 32	0.25	2	3.6	96.4
Ceftriaxone	≤ 0.06 – > 128	≤ 0.06	0.125	1.8	98.2
Cefoperazone-Sulbactam	≤ 0.06 – 16	0.25	1	0	100
Imipenem	≤ 0.06 – 0.5	0.125	0.25	0	100
Amikacin	0.25 – > 128	0.5	1	1.8	98.2
Colistin	0.25 – > 32	0.5	1	1.8	98.2
Tigecycline	0.25 – 16	1	1	1.8	96.4
***Klebsiella pneumonia* (ESBL +) (58)**					
Ceftobiprole	≤ 0.06 – > 128	> 128	> 128	93.1	6.9
Ceftazidime	2 – > 128	16	> 128	65.5	6.9
Ceftriaxone	0.25 – > 128	> 128	> 128	94.8	3.4
Cefoperazone-Sulbactam	0.5 – > 128	32	128	32.8	48.3
Imipenem	≤ 0.06 – 1	0.125	0.5	0	100
Amikacin	0.25 – > 128	1	> 128	10.3	89.7
Colistin	0.25 – > 32	0.5	1	3.4	96.6
Tigecycline	0.125 – 16	1	2	1.7	91.4
**b**					
**Carbapenem-resistant *Klebsiella pneumonia* (45)**					
Ceftobiprole	> 128 – > 128	> 128	> 128	100	0
Ceftazidime	16 – > 128	> 128	> 128	100	0
Ceftriaxone	4 – > 128	> 128	> 128	100	0
Cefoperazone-Sulbactam	64 – > 128	> 128	> 128	100	0
Imipenem	2 – 128	16	64	86.7	0
Amikacin	0.25 – > 128	> 128	> 128	60	40
Colistin	0.25 – > 32	0.5	2	8.9	91.1
Tigecycline	0.25 – 2	1	2	0	100
***Enterobacter cloacae* (49)**					
Ceftobiprole	≤ 0.06 – > 128	0.5	> 128	51	49
Ceftazidime	0.125 – > 128	2	> 128	36.7	59.2
Ceftriaxone	≤ 0.06 – > 128	1	> 128	46.9	51
Cefoperazone-Sulbactam	≤ 0.06 – > 128	4	> 128	22.4	65.3
Imipenem	≤ 0.06 – 4	0.5	4	12.2	87.8
Amikacin	0.5 – > 128	1	4	2	95.9
Colistin	0.25 – 4	0.5	2	4.1	95.9
Tigecycline	0.25 – 8	1	2	2	93.9
***Enterobacter aerogenes* (55)**					
Ceftobiprole	≤ 0.06 – > 128	≤ 0.06	> 128	20	80
Ceftazidime	0.125 – 128	1	32	29.1	70.9
Ceftriaxone	≤ 0.06 – > 128	0.25	> 128	27.3	70.9
Cefoperazone-Sulbactam	≤ 0.06 – 128	0.5	64	10.9	83.6
Imipenem	≤ 0.06 – 4	0.5	1	1.8	98.2
Amikacin	0.125 – 8	1	2	0	100
Colistin	0.125 – 4	0.5	1	1.8	98.2
Tigecycline	0.25 – 4	1	1	0	92.7
***Citrobacter freudii* (53)**					
Ceftobiprole	≤ 0.06 – > 128	≤ 0.06	> 128	35.8	64.2
Ceftazidime	0.25 – > 128	2	128	32.1	60.4
Ceftriaxone	≤ 0.06 – > 128	0.5	> 128	37.7	60.4
Cefoperazone-Sulbactam	0.125 – > 128	1	> 128	20.8	71.7
Imipenem	≤ 0.06 – 4	0.5	1	3.8	90.6
Amikacin	0.125 – > 128	2	4	1.9	98.1
Colistin	0.25 – 4	0.5	2	1.9	98.1
Tigecycline	0.25 – 4	0.5	1	0	98.1
**c**					
***Proteus mirabilis* (52)**					
Ceftobiprole	≤ 0.06 – > 128	≤ 0.06	> 128	34.6	65.4
Ceftazidime	≤ 0.06 – 2	≤ 0.06	0.25	0	100
Ceftriaxone	≤ 0.06 – 128	≤ 0.06	16	26.9	73.1
Cefoperazone-Sulbactam	0.25 – 4	1	4	0	100
Imipenem	≤ 0.06 – 2	0.5	1	0	94.2
Amikacin	0.5 – 32	2	8	0	98.1
Colistin	32 – > 32	> 32	> 32	100	0
Tigecycline	1 – 8	2	4	9.6	71.2
***Proteus vulgaris* (35)**					
Ceftobiprole	≤ 0.06 – > 128	32	> 128	82.9	17.1
Ceftazidime	≤ 0.06 – 64	≤ 0.06	1	5.7	94.3
Ceftriaxone	≤ 0.06 – > 128	≤ 0.06	32	20	74.3
Cefoperazone-Sulbactam	0.5 – 64	1	4	8.6	91.4
Imipenem	0.25 – 32	1	2	5.7	88.6
Amikacin	0.5 – 16	2	8	0	100
Colistin	32 – > 32	> 32	> 32	100	0
Tigecycline	0.5 – 4	2	4	0	88.6
***Morganella morganella* (53)**					
Ceftobiprole	≤ 0.06 – 64	≤ 0.06	32	20	80
Ceftazidime	≤ 0.06 – 32	0.125	2	7.3	92.7
Ceftriaxone	≤ 0.06 – > 128	≤ 0.06	8	16.4	80
Cefoperazone-Sulbactam	0.125 – 8	1	4	0	100
Imipenem	0.125 – 2	1	2	0	69.1
Amikacin	0.5 – > 128	2	8	1.8	98.2
Colistin	32 – > 32	> 32	> 32	100	0
Tigecycline	0.25 – 4	1	2	0	92.7
***Serratia marcescens* (53)**					
Ceftobiprole	≤ 0.06 – > 128	0.25	8	28.8	71.2
Ceftazidime	≤ 0.06 – 32	0.5	2	3.8	92.3
Ceftriaxone	≤ 0.06 – > 128	0.25	16	13.5	80.8
Cefoperazone-Sulbactam	0.5 – > 128	2	32	7.7	88.5
Imipenem	0.125 – 1	0.5	1	0	100
Amikacin	0.5 – > 128	2	8	1.9	96.2
Colistin	> 32 – > 32	> 32	> 32	100	0
Tigecycline	0.5–2	1	1	0	100

Ceftobiprole also had limited activity against *P. aeruginosa*, independent of susceptibility to carbapenems, with MIC_50/90_ 8/64- > 128 mg/L. Interestingly, for carbapenem-susceptible *A. baumanni*, 94.1% of strains were inhibited at ≤ 4 mg/L, showing the potency of ceftobiprole which was comparable to that of amikacin, cefoperazone-sulbactam, imipenem, colistin and tigecycline (MIC_50/90_ was 1/4, 1/2, 0.125/0.25 and 0.5/1 mg/L, respectively). However, for the carbapenem-resistant *A. baumanni*, ceftobiprole had negligible activity with a MIC_50/90_ of > 128 mg/L. For all *P. aeruginosa* and *A. baumannii*, colistin retained excellent in vitro activity (MIC_50/90_, 0.5–1/1–2 mg/L) (Table 6).

**Table 6:** Activity of ceftobiprole and comparator antimicrobial agents when tested against *Pseudomonas aeruginosa* and *Acinetobacter baumanni* isolated from China (mg/L)

**Antimicrobial agents**	**MIC Range**	**MIC**_**50**_	**MIC**_**90**_	**R%**	**S%**
**Carbapenem-susceptible* Pseudomonas aeruginosa* (19)**					
Ceftobiprole	1 – > 128	8	> 128	–	–
Ceftazidime	1 – > 128	4	> 128	10.5	68.4
Cefoperazone-Sulbactam	0.25 – > 128	4	64	10.5	84.2
Imipenem	0.25 – 4	0.5	4	0	84.2
Amikacin	0.5 – > 128	2	> 128	10.5	89.5
Colistin	0.5 – 2	1	2	0	100
**Carbapenem-resistant* Pseudomonas aeruginosa* (19)**					
Ceftobiprole	4 – > 128	8	64	–	–
Ceftazidime	8 – 64	16	64	36.8	26.3
Cefoperazone-Sulbactam	1 – 128	64	128	52.6	36.8
Imipenem	4 – 64	4	32	47.4	0
Amikacin	1 – > 128	2	16	5.3	94.7
Colistin	0.5 – 1	1	1	0	100
**Carbapenem-susceptible *Acinetobacter baumanni (*17)**					
Ceftobiprole	0.25 – > 128	0.5	4	–	–
Ceftazidime	2 – 64	8	8	5.9	94.1
Cefoperazone-Sulbactam	1 – 64	1	2	5.9	94.1
Imipenem	≤ 0.06 – 0 .5	0.125	0.25	0	100
Amikacin	0.25 – 16	1	4	0	100
Colistin	0.25 – 2	0.5	1	0	100
Tigecycline	0.25 – 1	0.25	1	0	100
**Carbapenem-resistant *Acinetobacter baumanni (*19)**					
Ceftobiprole	4 – > 128	> 128	> 128	–	–
Ceftazidime	32 – > 128	128	> 128	100	0
Cefoperazone-Sulbactam	16 – 128	64	64	52.6	36.8
Imipenem	4 – 128	16	32	89.5	0
Amikacin	1 – > 128	> 128	> 128	84.2	15.8
Colistin	0.5 – 2	0.5	2	0	100
Tigecycline	0.5 – 4	1	2	0	94.7

The MIC distribution of ceftobiprole is presented in Table 7a-b.

**Table 7:** The minimal inhibitory concentration (MIC) distribution of ceftobiprole when tested against different clinically isolated strains in China

**Organisms (no.)**	**Cumulative percentage of isolates at MIC (mg/L, %)**														
	** ≤ 0.015**	**0.03**	**0.06**	**0.125**	**0.25**	**0.5**	**1**	**2**	**4**	**8**	**16**	**32**	**64**	**128**	** > 128**
a															
MRSA (55)	0.0	0.0	0.0	0.0	5.5	27.3	80.0	100.0						–	–
MSSA (55)	0.0	0.0	0.0	0.0	1.8	69.1	98.2	100.0						–	–
MRCNS (40)	10.0	10.0	10.0	10.0	12.5	40.0	87.5	95.0	100.0					–	–
MSCNS (40)	5.0	5.0	15.0	32.5	65.0	97.5	100.0							–	–
*E. faecalis* (24)	0.0	0.0	4.2	4.2	25.0	66.7	91.7	91.7	91.7	91.7	91.7	91.7	100.0	–	–
*E. faecium* (23)	0.0	0.0	0.0	0.0	0.0	4.3	8.7	17.4	17.4	17.4	17.4	17.4	100.0	–	–
*S. pyogenes* (13)	100.0													–	–
*S. agalactiae* (14)	100.0													–	–
*S. mitis* (12)	58.3	58.3	75.0	75.0	75.0	83.3	83.3	100.0						–	–
PSSP (110)	24.5	28.2	40.0	55.5	74.5	98.2	100.0							–	–
PISP (25)	0.0	0.0	0.0	4.0	8.0	52.0	100.0							–	–
PRSP (13)	0.0	0.0	0.0	0.0	0.0	53.8	84.6	92.3	92.3	92.3	92.3	100.0		–	–
*H. influenzae* (53)	71.2	76.9	76.9	82.7	86.5	96.2	100.0							–	–
*M. catarrhalis* (49)	0.0	0.0	18.4	38.8	71.4	98.0	100.0							–	–
*E. coli (*ESBL-) (49)	–	–	59.2	89.8	91.8	95.9	95.9	100.0							
*E. coli* (ESBL +) (50)	–	–	0.0	0.0	0.0	0.0	4.0	4.0	4.0	4.0	6.0	8.0	8.0	8.0	100.0
b															
*K. pneumonia* (ESBL-) (56)	–	–	80.4	87.5	98.2	98.2	98.2	98.2	98.2	98.2	98.2	98.2	98.2	98.2	100.0
*K. pneumonia* (ESBL +) (58)	–	–	5.2	5.2	6.9	6.9	6.9	6.9	6.9	8.6	10.3	12.1	12.1	13.8	100.0
CR-KPN (45)	–	–	0.0	0.0	0.0	0.0	0.0	0.0	0.0	0.0	0.0	0.0	0.0	0.0	100.0
*E. cloacae* (49)	–	–	40.8	49.0	49.0	57.1	61.2	61.2	63.3	67.3	67.3	69.4	69.4	69.4	100.0
*E. aerogenes* (55)	–	–	54.5	74.5	80.0	83.6	85.5	85.5	87.3	87.3	87.3	87.3	87.3	89.1	100.0
*C. freudii* (53)	–	–	50.9	58.5	64.2	64.2	66.0	69.8	71.7	71.7	71.7	71.7	71.7	73.6	100.0
*P. mirabilis* (52)	–	–	55.8	65.4	65.4	65.4	65.4	67.3	67.3	67.3	69.2	69.2	69.2	71.2	100.0
*P. vulgaris* (35)	–	–	17.1	17.1	17.1	17.1	20.0	25.7	31.4	34.3	54.3	60.0	80.0	88.6	100.0
*M. morganella* (53)	–	–	65.5	78.2	80.0	83.6	85.5	87.3	87.3	87.3	87.3	96.4	100.0		
*S. marcescens* (53)	–	–	9.6	38.5	71.2	80.8	84.6	88.5	88.5	90.4	90.4	90.4	90.4	90.4	100.0
CS-PAE (19)	–	–	0.0	0.0	0.0	0.0	10.5	26.3	47.4	84.2	89.5	89.5	89.5	89.5	100.0
CR-PAE (19)	–	–	0.0	0.0	0.0	0.0	0.0	0.0	10.5	52.6	84.2	89.5	94.7	94.7	100.0
CS-ABA (17)	–	–	0.0	0.0	41.2	64.7	88.2	88.2	94.1	94.1	94.1	94.1	94.1	94.1	100.0
CR-ABA (19)	–	–	0.0	0.0	0.0	0.0	0.0	0.0	5.3	5.3	5.3	5.3	10.5	26.3	100.0

*MRSA* Methicillin-resistant *Staphylococcus aureus*, *MSSA* Methicillin-susceptible *Staphylococcus aureus*, *MRCNS* Methicillin-resistant Coagulase negative *Staphylococci*, *MSCNS* Methicillin-susceptible Coagulase negative *Staphylococci, PSSP Streptococcus pneumonia* with MIC of Penicillin ≤ 2 mg/L, *PISP Streptococcus pneumonia* with MIC of Penicillin = 4 mg/L, *PRSP Streptococcus pneumonia* with MIC of Penicillin ≥ 8 mg/L, *ESBL*- Extended spectrum β-Lactamases negative, *ESBL* + Extended spectrum β-Lactamases positive, *CR-KPN* Carbapenem-resistant *Klebsiella pneumonia*, *CS-PAE* Carbapenem-susceptible *Pseudomonas aeruginosa*, *CR-PAE* Carbapenem-resistant *Pseudomonas aeruginosa*, *CS-ABA Carbapenem-susceptible Acinetobacter baumanni*, *CR-ABA* Carbapenem-resistant *Acinetobacter baumanni*

## Discussion

As one of the limited new effective antibiotics approved for treating infection caused by resistant Gram-positive and Gram-negative bacteria, ceftobiprole has been evaluated in several studies in different medical centers around the world [7, 9, 10]. However, the published literature for its efficacy against contemporary clinical isolates from China is limited. In this study, we report on the activity of ceftobiprole and comparators against recent clinical isolates collected from hospitalized patients from 2016–2018 in China through the China Antimicrobial Surveillance Program. Our study suggest that ceftobiprole has high antibacterial activity against *Staphylococcus* (including MRSA) similar to the results from Europe and the United States [11]. We observed that MSSA strains were more susceptible to ceftobiprole than MRSA strains with one-fold lower MIC_90_. When compared to the earlier studies, the data reported in our study are comparable for ceftobiprole concerning the target gram-positive pathogens, such as *Staphylococcus*, *E. faecalis*, *Streptococcus*, supporting that ceftobiprole has a high susceptibility [9]. Ceftobiprole’s in vitro activity demonstrates potent binding against PBPs of gram-positive bacteria, including those with decreased β-lactam sensitivity, such as PBP2x and PBP2b in PRSP and, PBPa, which confers methicillin resistance to *S. aureus* strains [12].

Besides gram-positive bacteria, ceftobiprole also has good antibacterial activity against non-MDR gram-negative bacteria. Ceftobiprole exhibits a high affinity for PBPs in *Enterobacterales* but is labile to hydrolysis by common extended spectrum β-lactamases and carbapenemases. ESBL-negative *E. coli* and *K. pneumoniae*, MICs_50/90_ were both 0.03/0.06 mg/L in Europe and the USA, consistent with <  = 0.06/0.25 mg/L in the current study. Previous MIC results, including the SENTRY Antimicrobial Surveillance Program in the U.S. (2016) and in Europe (2015), demonstrated the potency of ceftobiprole against *Pseudomonas aeruginosa* (MIC_50/90_, 2/ >  = 16 mg/L) and had limited activity against *Acinetobacter spp.* (MIC_50/90_, >  = 16/ >  = 16 mg/L) [11, 13]. The data reported here showed a little difference in these two non-fermentative gram-negative bacteria with MICs_50/90_ were 8/ > 128 mg/L for carbapenem-susceptible *P. aeruginosa* and 0.5/4 mg/L for carbapenem-susceptible *A. baumanni*.

There were some limitations to our study. Firstly, ceftobiprole is approved for the treatment of community-acquired pneumonia and hospital-acquired pneumonia except for ventilator-associated pneumonia, but there is no relevant clinical disease information for the strains in our study. Secondly, there are a few strains of some *Streptococcus spp*, which may not fully demonstrate the antibacterial activity of cefpirome against such *Streptococcus spp*..

## Conclusion

Our study indicated that ceftobiprole showed potent in vitro activity against clinical significant pathogens including MRSA, MRCNS, *E. faecalis*, PRSP, *H. influenzae*, *M. catarrhalis*, ESBL-negative *Enterobacterales*, even carbapenem-susceptible *A. baumanni*, which could be a considerable choice for treating infections caused by those pathogens in healthcare facilities.

## Materials and Methods

### Clinical strains

A total of 1163 strains were selected randomly from 49 hospitals across China from 2016-to 2018, relying on the China Antimicrobial Surveillance Network (CHINET). Strains included methicillin-resistant *S. aureus* (MRSA, *n* = 55), methicillin-susceptible *S. aureus* (MSSA, *n* = 55), methicillin-resistant Coagulase negative *Staphylococci* (MRCNS, *n* = 40), methicillin-susceptible Coagulase negative *Staphylococci* (MSCNS, *n* = 40), *E. faecalis* (*n* = 24), *E. faecium* (*n* = 23), *Streptococcus pyogenes* (*n* = 13), *Streptococcus agalactiae* (*n* = 14), *Streptococcus mitis* (*n* = 12), *Streptococcus pneumonia* (MIC of Penicillin ≤ 2 mg/L, PSSP, *n* = 110), *Streptococcus pneumonia* (MIC of Penicillin = 4 mg/L, PISP, *n* = 25), *Streptococcus pneumonia* (MIC of Penicillin ≥ 8 mg/L, PRSP, *n* = 13), *Haemophilus influenzae* (*n* = 53), *Moraxella catarrhalis* (*n* = 49), *Escherichia coli* (ESBL-, *n* = 49), *Escherichia coli* (ESBL + , *n* = 50), *Klebsiella pneumoniae* (ESBL-, *n* = 56), *Klebsiella pneumoniae* (ESBL + , *n* = 58), *Enterobacter cloacae* (*n* = 49), *Enterobacter aerogenes* (*n* = 55), *Citrobacter freudii* (*n* = 53), *Proteus mirabilis* (*n* = 52), *Proteus vulgaris* (*n* = 35), *Morganella morganella* (*n* = 53), *Serratia marcescens* (*n* = 53), *Pseudomonas aeruginosa* (*n* = 38) and *Acinetobacter baumanni* (*n* = 36). Species identification was performed at the microbial laboratory of Huashan Hospital by the matrix-assisted laser desorption ionization-time-of-flight mass spectrometry (MALDI-TOF, Vitek MS; bioMérieux). *E. coli* ATCC 25,922, *P. aeruginosa* ATCC 27,853, *S. pneumoniae* ATCC 49,619, *H. influenzae* ATCC 49,766 and ATCC 49,247, *S. aureus* ATCC29213 and *E. faecalis* ATCC 29,212 were used as the quality control strains in antimicrobial susceptibility testing.

### Antimicrobial susceptibility testing

MICs were determined by the reference broth microdilution method recommended by the Clinical and Laboratory Standards Institute (CLSI) [14]. Ceftobiprole, linezolid, vancomycin, ampicillin, penicillin, oxacillin, ciprofloxacin, clindamycin, and erythromycin were tested for all Gram-positive bacteria; Ceftobiprole, ampicillin, ampicillin-sulbactam, cefuroxime, ceftazidime, ceftriaxone, ciprofloxacin, azithromycin, cefoperazone-sulbactam, imipenem, amikacin, colistin, and tigecycline were tested for Gram-negative bacteria as needed. Quality control and interpretation of the results were based on 2019 CLSI break-points for all the antimicrobial agents except tigecycline, for which CLSI criteria are not available [14]. Tigecycline MICs were interpreted using U.S. FDA MIC breakpoints for Enterobacterales (susceptible, ≤ 2 g/ml; resistant, ≥ 8 g/ml) (https://www.fda.gov/drugs/development-resources/tigecycline-injection-products).

## Data Availability

All data involved in this study are available from the corresponding author by email if needed.
